# Is Salivary S100B a Biomarker of Traumatic Brain Injury? A Pilot Study

**DOI:** 10.3389/fneur.2020.00528

**Published:** 2020-06-12

**Authors:** Damir Janigro, Keisuke Kawata, Erika Silverman, Nicola Marchi, Ramon Diaz-Arrastia

**Affiliations:** ^1^FloTBI Inc., Cleveland, OH, United States; ^2^Department of Physiology, Case Western Reserve University, Cleveland, OH, United States; ^3^Department of Kinesiology, School of Public Health, Program in Neuroscience, College of Arts and Sciences, Indiana University, Bloomington, IN, United States; ^4^Department of Neurology, University of Pennsylvania, Philadelphia, PA, United States; ^5^Cerebrovascular and Glia Research, Department of Neuroscience, Institute of Functional Genomics (CNRS/INSERM), University of Montpellier, Montpellier, France

**Keywords:** peripheral biomarkers, blood-brain barrier, saliva, mild traumatic brain injury, S100B

## Abstract

Traumatic brain injury (TBI) results in short and long-term disability neurodegeneration. Mild traumatic brain injury (mTBI) represents up to 85% of head injuries; diagnosis and early management is based on computed tomography (CT) or in-hospital observation, which are time- and cost- intensive. CT involves exposure to potentially harmful ionizing radiation and >90% of the scans are negative. Blood-brain barrier (BBB) damage is suspected pathological event post-TBI contributing to long-term sequelae and a reliable and rapid point-of-care test to screen those who can safely forego acute head CT would be of great help in evaluating patients with an acute mTBI. In this pilot study, 15 adult patients with suspected TBI (mean age = 47 years, range 18–79) and 15 control subjects (mean age = 33 years, range 23–53) were enrolled. We found that the average salivary S100B level was 3.9 fold higher than blood S100B, regardless of the presence of pathology. [S100B]_saliva_ positively correlated with [S100B]_serum_ (Pearson' coefficient = 0.79; *p* < 0.01). Salivary S100B levels were as effective in differentiating TBI patients from control subjects as serum levels (Control vs. TBI: *p* < 0.01; Serum ROC_AUC_ = 0.94 and Saliva ROC_AUC_ = 0.75). I These initial results suggest that measuring salivary S100B could represent an alternative to serum S100B in the diagnosis of TBI. Larger and confirmatory trials are needed to define salivary biomarker kinetics in relation to TBI severity and the possible roles of gender, ethnicity and age in influencing salivary S100B levels.

## Introduction

Traumatic brain injury (TBI) is common and it accounts for > 3 million emergency department visits per year in the United States alone (1). It has been widely recognized that there is need for improved clinical guidelines to diagnose TBI and to predict risk of downstream consequences. In particular, a consistent and rapid diagnosis of mild TBI (mTBI) based on blood analysis has been widely studied (2, 3). Serum S100B has been incorporated into Scandinavian guidelines for the management of acute mTBI, and serum GFAP and UCHL-1 have recently been cleared by the US Food and Drug Administration as an aid in the diagnosis of mTBI (2, 4). Although serum markers such as GFAP, UCH-L1 or S100B can assess for the presence of intracranial bleeding from TBI with an excellent negative predictive value using sensitive immunoassays, there is currently no translation of this technology to a saliva-based point-of-injury or point-of-care (POI/POC) device. Existing salivary tests for TBI are based on detection of pathologic forms of nucleic acids (5), but no POI/POC salivary test exists for the most common blood protein markers used in the evaluation of neurologic disorders.

Concussions, or mTBI, result in axonal disruption, neuroinflammation, and glial activation (6) which are major pathologic mechanisms underlying of cognitive and sensory impairments. In addition, mTBI can be associated with a rapid disruption of blood-brain barrier (BBB) integrity followed by eventual development of brain damage [e.g., (7–9)]. BBB disruption after TBI is also a risk factor for secondary brain hemorrhage (10–12). It is therefore important to detect early BBB disruption to predict the development of persistent post-concussion disability.

The peripheral BBB reporter S100B (13, 14) has been shown to correlate with several indices of BBB disruption including albumin coefficient (15), gadolinium enhancement (16–18), or CT scans (19). Most importantly, S100B increases also after a minor head injury (mTBI) and is useful to identify the absence of intracranial bleeding. In comparison to CT-based diagnosis of mTBI, S100B serum levels accurately identified negative intracranial bleeding, with a negative predictive value of 99% (20–23).

While the development of POI/POC devices is in the works, almost all POC prototypes use blood samples to measure biomarkers. Saliva is an attractive biological fluid, which has some advantages over blood testing in pre-hospital settings and direct to consumer home-based applications. Blood components, such as platelets or red blood cells, require a removal process before an analysis can be completed. In addition, blood samples cannot be drawn when paramedics or trained personnel are not present. In addition, risk of infection from blood products and the time and equipment necessary to separate blood components could make POC blood diagnostic challenging.

In an attempt to overcome this, we developed a prototype test for salivary S100B, which is being implemented in a laboratory medicine and in a POI/POC format. We report here the preliminary findings obtained by analyzing S100B in saliva of control subjects and mTBI patients. We enrolled patients with Glasgow Coma Scale (GCS) of 14–15 on admission to capture the mild forms of TBI which constitute the target population for our salivary S100B test.

## Methods

All patients (≥18 years old) presented to the emergency department at Penn Presbyterian Medical Center <6 h after an injury involving head trauma warranting a head CT for clinical evaluation, with a GCS of 14 or 15. Patients were excluded from the study if pregnant, in custody, and/or diagnosed with skull fractures. Written informed consent was obtained from the participants of the study for the publication of the identifiable data.

The Galveston Orientation and Amnesia Test (GOAT) was used to assess for a diminished capacity to consent. For this preliminary investigation, fifteen TBI subjects and fifteen control subjects were enrolled. Control subjects were defined as individuals who have no history of TBI in the past 6 months and do not display any neurological symptoms.

Demographic history, comorbidities, and clinical course of the injury, including GCS score, trauma mechanism, and loss of consciousness, were obtained at initial assessment by a treating physician, then confirmed by study staff when blood and salivary samples were collected.

Patients being treated in the hospital had an intravenous (IV) catheter placed for standard hospital monitoring. Blood was collected < 6 h from mTBI by draining off the IV whenever possible, otherwise collection was done by venipuncture. All control samples were collected by venipuncture. Whole blood was processed for serum, within 30 min of collection. After centrifugation, serum was transferred into cryovials labeled with a unique subject ID number and stored at −80C in 0.5 ml aliquots.

Saliva was collected with a commercially available collection system (Salivette™) at time of blood draws. The subjects were asked to chew a plain cotton roll for 1 min to stimulate salivation. The rolls with the absorbed saliva were then centrifuged to removed food remnants, insoluble material, and cell debris. The resulting supernatant was stored in 0.5 ml aliquots at −80C.

S100B was measured, in both saliva and serum, by chemiluminescence and automated sandwich ELISA (LIAISON; Diasorin, Stillwater, MI) (24). The Limit of Detection is 0.02 ng/mL, and the lower-upper range is 0.02–30 ng/mL. The inter- and intra-coefficient of variation are 9.4 and 7.2%, respectively.

To assess the significance of differences between groups we used Minitab 19 and linear regression/ Mann-Whitney tests. Because clinical data are not usually normally distributed, they are also reported as median and interquartile ranges (**Table 2**). Outliers were removed in Origin 2019 Pro by Grubbs test. ROC were obtained with JMP 14 (SAS).

## Results

**Figure 1:**
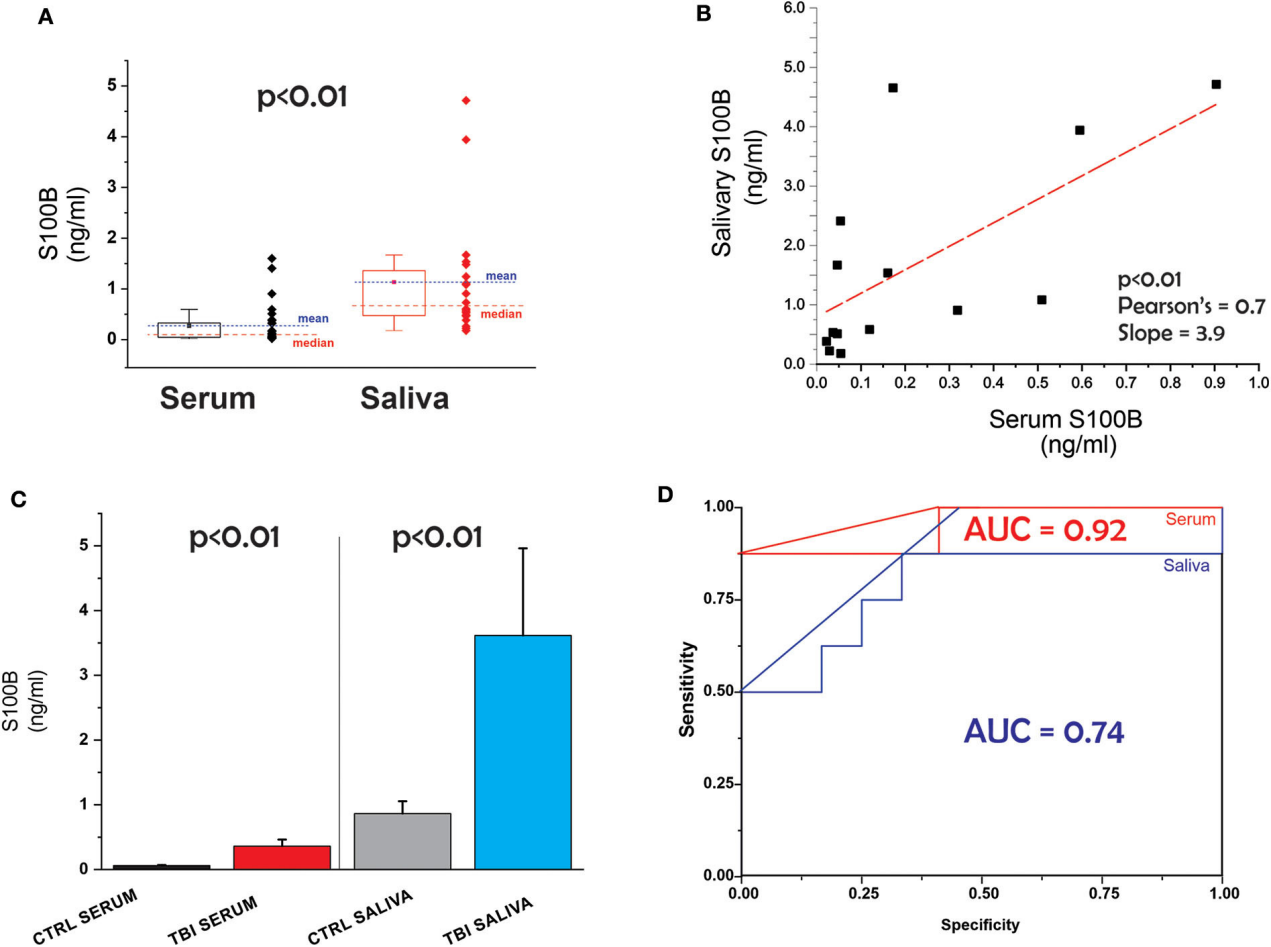
**(A)** Serum and salivary levels of S100B in all subjects. *Red* and *blue* symbols differentiate between control and TBI. **(B)** Correlation between salivary and serum S100B. **(C)** Comparable predictive value for TBI of saliva and serum; **(D)** ROC curves for salivary and serum S100B.

The patients' demographic and clinical characteristics are shown in Table 1. Comparison of all data points (serum vs. saliva, Figure 1A) revealed that levels of the biomarker S100B are higher in saliva than blood. Figure 1B shows the correlation between serum and salivary S100B levels. Line slope is 3.9, the Adj. R-Square 0.38, Pearson's coefficient 0.7 and the p value *p* < 0.01.

**Table 1:** Demographic and clinical characteristics of the patients and controls.

**ID**		**Gender**	**Ethn**.	**Age**	**GCS at admission**	**Time to collection of samples**	**CT findings (0,1)**	**CT descriptive**	**Symptoms amnesia, vomiting, et**.
SB-1001	CONTROL	Male	Cauc.	29	NA	NA	NA	NA	NA
SB-1002	CONTROL	Male	Cauc.	25	NA	NA	NA	NA	NA
SB-1007	CONTROL	Male	Cauc.	29	NA	NA	NA	NA	NA
SB-1009	CONTROL	Male		53	NA	NA	NA	NA	NA
SB-1012	CONTROL	Male	Cauc.	31	NA	NA	NA	NA	NA
SB-1020	CONTROL	Male	AA	23	NA	NA	NA	NA	NA
SB-1021	CONTROL	Female	Cauc.	32	NA	NA	NA	NA	NA
SB-1024	CONTROL	Male	Cauc.	37	NA	NA	NA	NA	NA
SB-1025	CONTROL	Female	Cauc.	30	NA	NA	NA	NA	NA
SB-1026	CONTROL	Male	Cauc.	35	NA	NA	NA	NA	NA
SB-1027	CONTROL	Male	Cauc.	28	NA	NA	NA	NA	NA
SB-1028	CONTROL	Female	Cauc.	27	NA	NA	NA	NA	NA
SB-1029	CONTROL	Male	Cauc.	41	NA	NA	NA	NA	NA
SB-1030	CONTROL	Female	Cauc.	26	NA	NA	NA	NA	NA
SB-1014	CONTROL	Male	AA	53	NA	NA	NA	NA	NA
SB-1003	TBI	Male	Cauc.	77	14	4:20	0		LOC
SB-1004	TBI	Male	Cauc.	76	15	4:19	1	Acute left frontal subarachnoid hemorrhage	AOC, LOC, PTA
SB-1005	TBI	Female	Cauc.	61	15	4:51	0		PTA, AOC
SB-1006	TBI	Female	AA	21	15	3:58	0		mild PTA, LOC
SB-1008	TBI	Male	AA	29	15	5:02	0		PTA, Dizziness, Headache
SB-1010	TBI	Female	AA	55	14	2:43	0		LOC, PTA
SB-1011	TBI	Male	AA	18	15	4:58	0		LOC, PTA
SB-1013	TBI	Female	AA	33	15	3:40	0		LOC, Headache
*SB-1015	TBI	Male	Cauc.	25	15	5:00	0		LOC, Headache
*SB-1016	TBI	Male	AA	41	15	2:38	0		LOC
*SB-1017	TBI	Female	AA	68	15	5:02	1	Acute right extra axial hemorrhage hematoma	LOC, nausea, vomiting
SB-1018	TBI	Male	AA	58	15	1:46	0		LOC
SB-1019	TBI	Male	AA	39	15	4:55	0		Headache
*SB-1022	TBI	Female	AA	28	15	2:39	0		LOC, headache
SB-1023	TBI	Male	AA	79	15	3:34	0		Headache

The ^*^ points to outliers detected by Grubbs test. Cauc, Caucasian; AA, African-American; AOC, LOC, Alteration, loss of consciousness; PTA, post-traumatic amnesia.

Next, we tested the hypothesis that S100B in saliva is as effective as blood S100B in detecting TBI vs. control. The results are shown in Figure 1C. We used a non-parametric test (Mann-Whitney) to determine the statistical significance of the observed increases in salivary and blood S100B after a diagnosis of mTBI. Significantly elevated levels of both salivary and blood S100B were measured in mTBI patients compared to controls (*p* < 0.01) (Table 2). ROC curves for saliva and serum are shown in Figure 1D. The two curves were not statistically different (*p* > 0.05). Finally, we performed an analysis of variance to detect if potential confounders (ethnicity, age, and gender) other than TBI had influenced blood or serum levels of S100B. We conducted a multivariate linear regression that included saliva and blood S100B (continuous variable) as outcomes, group as fixed effect/predictor, and age/race/sex as covariates. A limitation of our study is the unequal repartition of AA and Caucasian subjects among control and TBI cohorts. The model revealed that these covariates did not have significant influence on the outcomes. we report the results without covariates and also with race (we prioritized race, given the small sample size) as a covariate. Both models showed significant group differences in blood (without covariate β = −0.301, *SE* = 0.074, *p* = 0.001; with race β = −0.230, *SE* = 0.110, *p* = 0.050) and saliva (without covariate β = −2.768, *SE* = 0.986, *p* = 0.011; with race β = −3.321, *SE* = 1.492, *p* = 0.038).

**Table 2:** Data reported as median and interquartile values.

		**N**	**Mean**	**Standard Error**	**Standard Deviation**	**Coefficient of Variation**	**Minimum**	**Quantile**	**Median**	**Maximum**
**Blood**	**CONTROL**	15	0.058	0.008	0.032	54.620	0.022	0.037	0.047	0.122
	**TBI**	14	0.502	0.129	0.483	96.270	0.037	0.170	0.353	1.600
**Saliva**	**CONTROL**	15	0.849	0.159	0.615	72.430	0.223	0.466	0.584	2.411
	**TBI**	8	3.620	1.340	3.800	105.150	0.180	0.950	2.740	11.920

## Discussion

To our knowledge, this is the first study demonstrating a correlation between salivary and blood S100B levels in adults with mTBI. The results have shown that: (1) salivary levels of the astrocytic protein S100B are higher than those measured in serum; (2) the diagnostic properties of S100B in blood are similar to those in saliva; (3) a correlation exists between salivary and blood levels of S100B in control and post-TBI conditions.

A crucial aspect of TBI diagnostics is the fact that although serum markers such as GFAP, UCH-L1 or S100B already rule out clinically important concussion sequelae with an excellent negative predictive value and a low limit of detection in laboratory-based approaches, there is currently no translation of this technology to a saliva-based POI or POC device. The POI/POC platforms currently in development use blood where platelets, leukocytes and red blood cells require a cumbersome removal process prior to protein analysis. The equipment used to process serum/plasma and measure biomarkers in blood is not always available. Finally, a saliva-based POI/POC device would solve several of the problems associated with blood diagnostics in TBI or other neurological diseases. The development of new diagnostic methods to rapidly screen for TBI related brain damage is of utmost significance for head-contact sports, military and emergency head trauma settings. The use of saliva in place of serum or plasma samples could further streamline the diagnostic routine, simplifying the execution of testing.

Saliva is an important biofluid for evaluation of health and disease in human subjects. The use of saliva for diagnostics has advantages, but an obstacle in the progression of this field has been lack of a detailed understanding of how dynamic passage of biomarkers from blood into saliva occurs. To substantiate the use of salivary diagnostic approach, several research groups have reported their pilot data. For example, Di Pietro et al. (25) recruited small sample size (*n* = 6 concussion, *n* = 6 controls) and screened 800 human microRNA expression levels in saliva samples. Five microRNAs were significantly elevated in the concussion group compared to the control group, yet these genes were non-brain specific. Subsequently, (26) identified 5 panels of microRNA, which are related to neuronal functional integrity, to be significantly elevated in saliva in pediatric concussion patients (avg. age of 14 years). The salivary microRNA measure was particularly useful in detecting prolonged concussion recovery, with 85% accuracy in detecting patients with prolonged concussion symptoms from their acute symptomatic counterparts. It is worth also noting that whether measured in blood or saliva, S100B is an indicator of blood-brain barrier disruption (23, 27). Finally, we have recently published a paper describing the pharmacokinetic aspects of biomarkers' origin, fate and distribution (28). This was limited to traditional body fludis (blood, urine). We now expanded this to encompass salivary biomarkers (13).

To highlight the prospective advantage of various biomarkers in saliva-based diagnostics, we recently developed a computer algorithm to reproduce the passage of small molecular weight protein from blood into saliva (13). This program was originally designed as a “physiologically-based pharmacokinetic model to describe the distribution of brain-derived biomarkers in blood” (28). Its main structure was expanded to include a new compartment, namely an idealized salivary gland receiving its vascular supply by the external carotid. The venous output was approximated by jugular vein branches. To approximate the combined contribution of protein extravasation along transcellular and paracellular pathways crossing capillary endothelial cells and salivary gland epithelia, we used a simple mass transfer equation to quantify the process. While this approach gave an insight into the kinetics of passage of protein from blood to saliva (13), it did not predict significantly higher S100B levels in saliva vs. blood. A question that will be answered by future studies thus relates to the several-fold change in S100B levels in saliva vs. serum (Figure 1A).

The main limitation of this report is the small numbers of TBI cases. Larger studies in children and adults are being performed in the US and Europe. Regardless of its preliminary nature, this initial results provides evidence supporting the continuous studying of salivary S100B in TBI diagnostics. Also, we believe that the implementation of salivary S100B into clinical guidelines could be cost and time saving. An additional limitation is the use of normal subjects as controls: a population of non-trauma victims (e.g., orthopedic emergency room patients) may have been more appropriate. It is important to underscore that in a small pediatric study (29), salivary S100B was not increased in polytrauma no-TBI patients.

## Data Availability Statement

The datasets generated for this study are available on request to the corresponding author.

## Ethics Statement

The studies involving human participants were reviewed and approved by IRB University of Pennsylvania. The patients/participants provided their written informed consent to participate in this study.

## Author Contributions

DJ directed the study. RD-A and ES performed all the clinical studies. KK and NM assisted in data analysis and manuscript preparation. RD-A and DJ led the overall effort.

## Conflict of Interest

DJ is the founder of FloTBI, Inc. a company developing the salivary test presented herein. The remaining authors declare that the research was conducted in the absence of any commercial or financial relationships that could be construed as a potential conflict of interest.
